# When sex doesn’t sell to men: mortality salience, disgust and the appeal of products and advertisements featuring sexualized women

**DOI:** 10.1007/s11031-017-9615-9

**Published:** 2017-05-18

**Authors:** Seon Min Lee, Nathan A. Heflick, Joon Woo Park, Heeyoung Kim, Jieun Koo, Seungwoo Chun

**Affiliations:** 1 Institute for Business Research and Education, Korea University, Seoul, Republic of Korea; 2 School of Psychology, University of Lincoln, Lincoln, UK; 3 Dongkuk Business School, Dongguk University, Seoul, Republic of Korea; 4 Hyupsung University, Gyeonggi-Do, Republic of Korea; 5 College of Business Administration, Pukyong National University, Busan, Republic of Korea

**Keywords:** Mortality salience, Sex-appeal, Disgust, Advertisements, Terror management

## Abstract

Although men typically hold favorable views of advertisements featuring female sexuality, from a Terror Management Theory perspective, this should be less the case when thoughts of human mortality are salient. Two experiments conducted in South Korea supported this hypothesis across a variety of products (e.g., perfume and vodka). Men became more negative towards advertisements featuring female sexuality, and had reduced purchase intentions for those products, after thinking about their own mortality. Study 2 found that these effects were mediated by heightened disgust. Mortality thoughts did not impact women in either study. These findings uniquely demonstrate that thoughts of death interact with female sex-appeal to influence men’s consumer choices, and that disgust mediates these processes. Implications for the role of emotion, and cultural differences, in terror management, for attitudes toward female sexuality, and for marketing strategies are discussed.

## Introduction

Using sexuality—and in particular female sexuality—to sell products has a pronounced history that continues today. In 2003, 27% of advertisements featured female sexualization, up from 15% in 1983 (Reichert et al. 2012). This popularity occurs across media types and cultures (Nelson and Paek 2005). Yet, despite their prevalence, research shows that these advertisements typically increase the appeal of products for men, but not for women (Baumeister and Twenge 2002; Dahl et al. 2009; Ford et al. 1991; Griffitt and Kaiser 1978; LaTour 1990; LaTour and Henthorne 1993; Richmond and Hartman 1982; Sengupta and Dahl 2008; Wyllie et al. 2014).

Research based on Terror Management Theory (TMT) (Greenberg et al. 1986) provides insight into when men might be less inclined to be drawn to advertisements featuring female sexuality. Specifically, mortality salience causes men, but not women, to become more negative towards sexually alluring women (Landau et al. 2006; Morris and Goldenberg 2015). Advertismeents are more effective when product endorsers are viewed favorably (Goldsmith et al. 2000; Martin et al. 2002; Petty et al. 1983; Vakratsas and Ambler 1999), In turn, we hypothesized that for men (but not women) thoughts of human mortality should reduce the effectiveness of these advertisements.

### Terror management theory

From the perspective of TMT (Greenberg et al. 1986), there is a basic psychological conflict between wanting to live and knowing that ultimately, life will end. This creates a potentially massive amount of anxiety that could, conceivably, manifest itself as terror. Humans, however, maintain psychological stability via a dual, largely symbolic, system of cultural worldviews and self esteem. A cultural worldview provides a shared conception of reality that makes it possible for individuals to live with a sense of order, meaning, stability and, ultimately, symbolic immortality (e.g., contributing to something that outlives the self) and/or literal immortality (i.e., afterlife belief). Self-esteem serves as a gauge by which someone is living up to their cultural worldview, and hence, by which this potential anxiety is managed.

Hundreds of studies support the role of cultural worldviews and self-esteem in protecting people psychologically when death is salient (for review see Burke et al. 2010; or; Solomon et al. 2004). When reminded of human mortality, people behave and think in ways more consistent with their cultural values. In Western cultures, this releates to increased hostility in various forms towards worldview critics and out-group members (Greenberg et al. 1990; McGregor et al. 1998), and in Eastern cultures this relates to increased concern for others (Feng et al. 2016; Ma-Kellams and Blascovich 2011). Worldview defense and self-esteem are also associated cross-culturally with better mental health when mortality is salient, including the belief that life is meaningful (Simon et al. 2008), feelings of hopefulness, and stability (as opposed to increases) of negative affective states (Routledge et al. 2010; Wisman and Heflick 2016; Wisman et al. 2015). Thus, the TMT tenets that cultural worldviews and self-esteem help people cope with thoughts of their own mortality are well supported cross-culturally.

### Terror management theory, sexuality and denying human-animal similarities

But what do thoughts of death specifically have to do with people’s beliefs about, and comfort with, human sexuality? Building upon TMT, Goldenberg and colleagues (e.g., Goldenberg et al. 2001; Roberts et al. 2002) have argued that one way in which people cope with thoughts of death is by psychologically transforming themselves into symbolic, non-mortal beings. A key aspect of this is to deny—or at least psychologically distance from- the animalistic aspects of one’s own own existence. Certain aspects of the body and of sexuality, such as sweating, defecating, genital pleasure, and reproduction, are inherently shared between humans and non-human animals, and, ultimately, the physical body decays and dies. As such, one’s sexuality and one’s own body, unless “wrapped up” in a dressing of cultural symbols that provide it with meaning and value that separates it from, or covers up, the animality, become problematic when thoughts of death are salient.

Several studies conducted in Western cultures are consistent with the theorizing that humans distance from their animality when mortality is salient. Thoughts of death reduce agreement with essays emphasizing human-animal similarities relative to essays arguing for human- animal uniqueness (Goldenberg et al. 2001), reduce belief that humans evolved from other animals (Tracy et al. 2011), and heighten belief that one’s ingroup is distinct from animals (Vaes et al. 2010). Belief in evolution was not altered by mortality salience, however, when it was framed as having a unique advantage for humans relative to other animals. Making death salient also reduces interest in the physical (less uniquely human) aspects of one’s own sex life, such as pure genital pleasure (Goldenberg et al. 1999), but not in the uniquely human aspects of sex, such as love and romance (Goldenberg et al. 2002), and reduces usage of the physical body (Goldenberg et al. 2008), even when the experience is pleasurable (Goldenberg et al. 2006) or healthy (e.g., breast-cancer screening; Goldenberg et al. 2008). This body of research indicates that people distance from their own animality, including their own body and sexuality, when mortality is salient, unless these aspects have been framed in a way that maintains their unique humanness.

### Terror management, men, and distancing from female sexuality

Although mortality salience motivates a general psychological distancing from animality and one’s own sexuality, Goldenberg and colleagues (Goldenberg et al. 2000; Goldenberg and Roberts 2004; Landau et al. 2006) have argued that female sexuality poses a unique psychological threat to men when mortality is salient. Specifically, they argue that female sexuality and the female body are associated with animality and creatureliness. Men, being sexually attracted by women’s bodies, are—according to this perspective—motivated to distance from, and derogate, female sexuality when mortality is salient. This functions to assuage concerns that they are not themselves animalistic, which are heightened by mortality salience. In turn, from this perspective, women should not denigrate sexualized women due to mortality concerns (they do not have the same level of sexual arousal towards women and their attraction is not as body oriented), and men and women are not likely to denigrate sexualized men when mortality is salient (men when sexualized are not associated with animality).

A wide variety of research is consistent with the building blocks of this perspective. Sexualized women are perceived of as more animalistic than non-sexualized women at both explicit and implicit levels, and this is not the case for males when sexualized (Morris et al. 2017; Vaes et al. 2011). Men also have a greater body-oriented sexual attraction relative to women’s sexual attraction (Ford et al. 1991; Richmond and Hartman 1982), and greater sexual arousal in general (Dahl et al. 2009; Sengupta and Dahl 2008). Further, although no research has directly looked at derogating, and distancing from, sexualized women as a means of asserting one’s own humanness, people tend to exaggerate their uniqueness from people with undesirable traits, particularly when concerned that they themselves might be perceived as having those traits (Schimel et al. 1999). As such, there is a solid body of evidence consistent with the idea that men should distance from sexualized women when thinking about death, but that women should not.

Indeed, experimental evidence is very consistent with this theorizing; men evaluate sexualized women more negatively, and distance from them more, when mortality is salient (Landau et al. 2006; Morris and Goldenberg 2015). In Landau et al. (2006), men who were assigned to the mortality salience (vs. control) condition evaluated sexually promiscuous women in images as less attractive (Study 1). They also reported reduced sexual intentions toward a sexually attractive woman after having a real interaction with her (Study 2), and in conjunction with a lust prime, reported greater acceptance of a woman being physically abused. Thoughts of death did not cause men or women to denigrate non-sexualized women (Study 3). Furthermore, women’s atitudes toward sexually alluring men and sexually alluring women did not change when mortality was salient across several studies. In other studies, men were less likely to be attracted to sexualized women in advertisements when mortality was salient (Morris and Goldenberg 2015).^1^

### Mortality salience and consumerism

No research we know of has investigated the effects of thinking about one’s own death on evaluations of sex-appeal advertisements, or the desire to purchase products sold using female sexuality. However, several researchers (e.g., Ferraro et al. 2005) have demonstrated that thoughts of death can impact consumer behavior in ways consistent with TMT. Indeed, mortality salience has been found to increase the motivation to buy products (Das et al. 2014), especially when they are associated with one’s own worldview (Rangan et al. 2015).

### From endorser views to advertisement effectiveness

People’s beliefs, attitudes, and feelings toward endorsers play an important role in the decision buy a product or endorse a cause, with favorable views—such as believing that endorsers can be trusted, liking them and not dehumanizing them—relating to heightened willingness to purchase associated products or donate money to associated charities (Bongiorno et al. 2013; Goldsmith et al. 2000; Martin et al. 2002; Petty et al. 1983; Vakratsas and Ambler 1999). Companies recognize the importance of who endorses their products; Tiger Woods, for instance, according to some researchers increased golf ball sales for Nike by $103 million over 10 years (Chung et al. 2013). On the flip-side, negative information about product endorsers—such as moral scandals—reduces positive feelings towards a company (Till and Shimp 1998) and lowers stock prices (Bartz et al. 2013). As such, negative perceptions of a person in an advertisement should relate to more negative attitudes towards the advertisement itself, and as an extension reduce people’s willingness to purchase products.

### Disgust as a potential mediator

Disgust is an emotion that may mediate men’s negative reactions toward sexualized advertisements when mortality is salient. Disgust is a condemning emotion that emerges when others violate body-related expectations (Graham et al. 2009; Russell and Giner-Sorolla 2012). Disgust sensitivity predicts some responses to mortality salience (Kelley et al. 2015), and is heightened by mortality salience in conjunction with bodily aspects that are perceived as animalistic (Goldenberg et al. 2001). Men’s sexual attraction to women is highly body-oriented (Ford et al. 1991), and thoughts of death motivate people to see themselves as more distinct from other animals (Goldenberg et al. 2000). As such, we predicted that disgust might mediate the relationship for men between mortality salience and negative reactions to advertisements featuring female sexualization.

### Novelty and contributions

These studies make several potential contributions. First, few studies have identified conditions that weaken the appeal of sexual advertising for men. In addition, scarce research has explored how different media contexts (e.g., contents of TV program vs. newspaper articles) influence individuals’ reactions to sex appeal advertisements, even though this topic is widely considered to be important in advertising research (Meyers-Levy and Tybout 1997; Wang and Calder 2006). This study also would contribute to a growing body of research on how thoughts of death impact perceptions of sexualized women (e.g., Goldenberg et al. 2000; Goldenberg and Roberts 2004) by (a) testing if the negative effects extend beyond the woman herself, and into the products and brands she is endorsing, and (b) testing a proposed mediator for this effect. Moreover, if our hypothesis is true regarding disgust, it would add to accumulating evidence that emotion, and in particular specific emotions (as opposed to broad measures of negative and positive affect) mediate some responses to mortality salience (e.g., Lambert et al. 2014). And finally, there are many situations in which sexualized advertisements might appear in either temporal (a commercial or news topic prior to or after the sexualized advertisement) or physical proximity (two online advertisements sharing space next to a news article), making this research question of practical importance for businesses and marketers as well.

## Overview and hypotheses

In two experiments using different forms of media, we tested the hypothesis that men’s, but not women’s, reactions to advertising with female sexual appeal will become more negative when thinking about mortality. The first study was conducted to test responses to ads with a sexually alluring female model for perfume and vodka brands when mortality was salient (vs. a control condition). The second study used a similar design, but with an advertisement for a hamburger brand, which is presumably associated less with sexuality. In Study 2, disgust was also measured as a potential mediator of the proposed findings. We hypothesized, based on TMT, that men, but not women, would become more negative towards products and advertisements that utilize female sex-appeal, and that these effects would be mediated by disgust.

## Study 1

### Method

#### Participants

Two hundred forty four undergraduate students (females = 131, males = 113) from a university in Seoul, South Korea participated in exchange for course credit. All participants were native Korean speakers, and all materials were presented in the Korean language.

#### Procedure and materials

For each session, a group of participants (between 20 and 30) were instructed to sit in front of individual computers as they entered a computer lab within the university. At the beginning of each session, participants were told that they would view and evaluate a video clip and advertisements.

The design of the experiment was a 2 (MS: Death vs. Control) × 2 (Participant Gender: Male vs. Female) × 2 (Sex appeal: Yes vs. No) between-subjects factorial design. Participants were randomly assigned to watch either a death-related video or an anxiety-inducing extreme sports video, which served as an aversive control condition to parallel the potential negativity induced by death thoughts. To elicit death thoughts, the death-related video contained several events (e.g., September 11) causing many deaths. In the control condition, the video was about several extreme sports (e.g., bungee jumping) inducing thrilling and anxious feelings. The length of the video was approximately 5 min in each condition. After watching the video, participants performed a word completion task as a manipulation check to assess thoughts of death. Participants were given ten word fragments, all of which could be completed with either a neutral or a death-related Korean word according to the primed thoughts [based on Greenberg et al. 1994; e.g., DE _ _ (dead vs. deer), FU_ _ (funeral vs. future)]. All words were translated into either 2- or 3-letter Korean words which share one syllable (see “Appendix”). Participants were asked to fill in the remaining part(s): one or two letter(s) in either the front or the last (see “Appendix” for details). Then, they responded to some filler questionnaires measuring mood (a Korean translation of the PANAS; Watson et al. 1988) as in previous research (e.g., Greenberg et al. 2000). These filler questionnaires served as a delay between the mortality salience manipulation and the dependent measures to ensure that thoughts of death were activated, but no longer conscious, and also to test if general mood was associated with any potential findings (which we did not expect; see Arndt et al. 2004, for discussion of proximal vs. distal effects in terror management; see; Lambert et al. 2014, for discussion of the role of affect in responses to mortality salience).

**Fig. 1:**
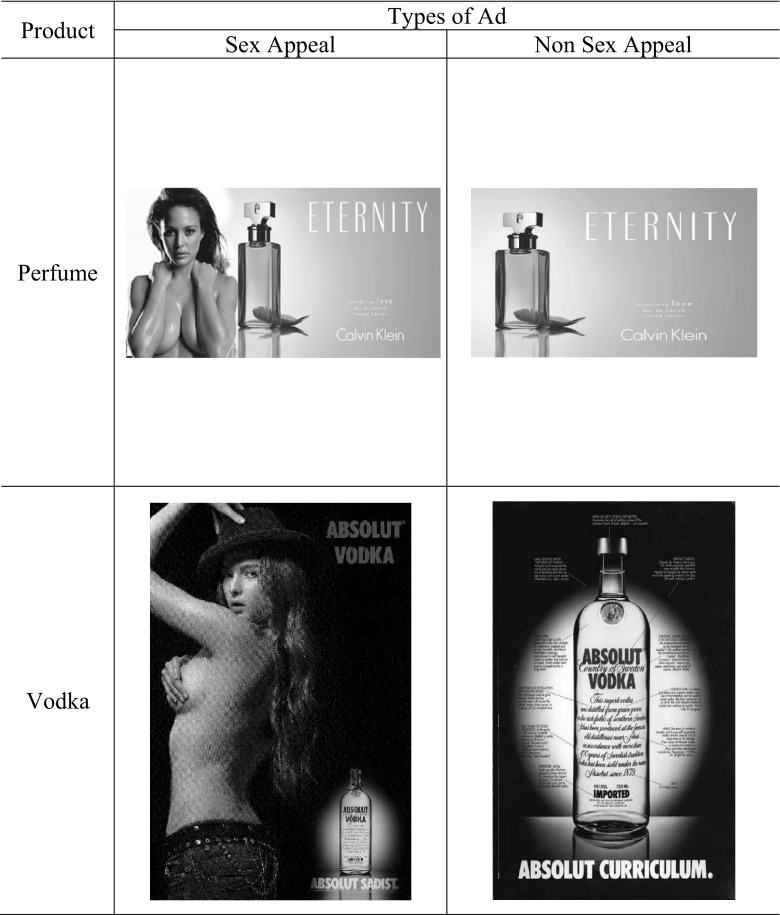
Study 1 advertising stimuli

Then, participants were randomly assigned to either the sex-appeal advertising condition or non sex-appeal advertising condition (see Fig. 1). Two types of product (perfume and vodka) were used for the stimuli. Each sex appeal advertisement for each product featured a female model in a sexual pose with the product. The control advertisements featured a product without any model. In this experiment, participants were exposed to exclusively either two sex appeal ads or two control ads (not a combination of a sex appeal and a control ad). Exposure time for each ad was controlled by setting 20 s for the page containing an ad to be advanced to the next page in the online survey. After reviewing the first advertisement, each participant responded to a questionnaire containing our dependent variable measures for the first advertisement exposed to them. Then, they repeated this process for the second ad. The presentation order of the ads and dependent variables was counterbalanced across participants.

The two dependent variables were assessed using identical items from past research (Sengupta and Dahl 2008). First, advertisement attitudes were measured on three seven-point scales (1 = *poor*/*bad*/*dislike*; 7 = *excellent*/*good*/*like*; α = 0.88). Next, purchase intentions toward the featured products in the ads were measured with two items on a seven-point scale (1 = *do not intend to recommend*/*purchase the product*; 7 = *intend to recommend*/*purchase the product*; α = 0.95). Scores were summed and averaged across products for all dependent variables.

After the dependent variables (for both products), participants were asked to rate the perceived sexuality of the ads as well as relevance of the products to them. For perceived sexuality, we used two items (sexual and sexy) on an 11-point scale anchored at 1 = “*not at all*,” 11 = “*extremely*,” by adopting from Sengupta and Dahl (2008). All dependent variables were summed across products. Finally, after answering demographic measures, participants were debriefed, thanked, and dismissed.

### Results

#### Manipulation checks

To check that the video pertaining to mortality was effectively inducing higher levels of death related thoughts, an index of death thought accessibility was created by averaging the total number of death-related words each participant completed. Indeed, participants in the mortality-video condition completed more word fragments with death-related words than those in the control condition [*M*_death_ = 2.47 vs. *M*_control_ = 0.82; *F*(1, 242) = 89.23, *p* < .001]. This indicates that the mortality salience manipulation was successful.

We also tested if our intended “sex-appeal” advertisements were deemed to differ in sexuality from the control advertisements. The indexes of the sexuality measure for the two products were reliable (Perfume: α = 0.84, Vodka: α = 0.89). As planned, participants rated the sex appeal advertisements to be more sexual than those in the control condition [Perfume: *M*_sex−appeal_ = 6.52 vs. *M*_control_ = 3.99; *F*(1, 242) = 91.40, *p* < .001; Vodka: *M*_sex−appeal_ = 5.88 vs. *M*_control_ = 2.77; *F*(1, 242) = 53.21, *p* < .001].

#### Affect

Participants reported a difference between mortality salience conditions for negative mood on the PANAS measure [*M*_death_ = 3.16 vs. *M*_control_ = 2.72; *F*(1, 242) = 9.11, *p* = .003]. However, the analysis did not reveal any difference between MS conditions for positive mood, *F*(1, 242) = 1.88, *p* = .17. Therefore, negative mood was statistically controlled in the following analyses to test what effect, if any, this difference may have played in our planned analyses. Though past work consistently has shown that broad (summed) measures of negative and positive affect do not mediate mortality salience effects (Burke et al. 2010), it has not utilized this specific manipulation of death thoughts.

#### Ad attitudes

We hypothesized that male participants would be more unfavorable towards advertisements featuring a sexualized woman model when thinking about mortality, whereas female participants’ reactions toward the same advertising would not be altered by mortality salience. To test this, a three-way ANCOVA (with negative mood as a covariate) was conducted for both the perfume and the vodka advertisements. Please note that effects that are not specifically listed for each analysis did not approach significance (*ps* > 0.15).

**Fig. 2:**
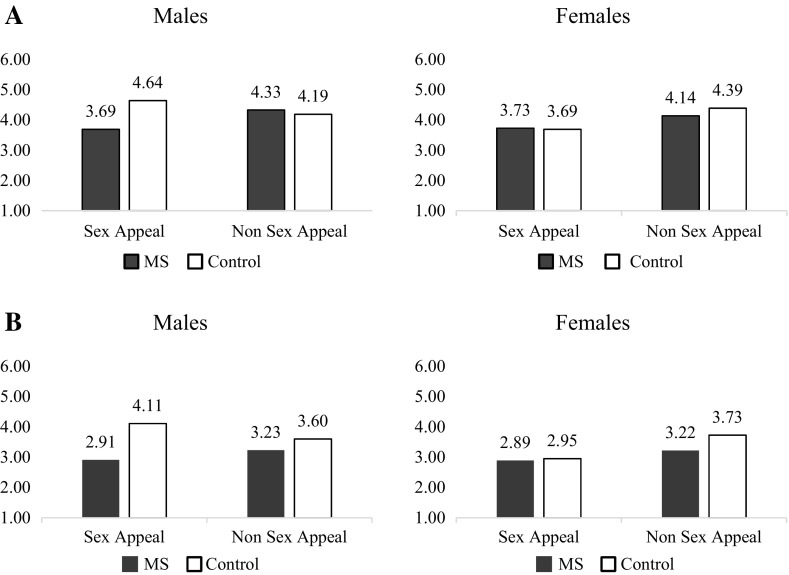
Sex appeal ad Attitudes (**a**) and intention to purchase (**b**) in Study 1

For attitudes toward the advertisements, there was a significant main effect for gender [*F*(1, 235) = 4.15, *p* = .04], as well as sex appeal [*F*(1, 235) = 8.92, *p* < .01], and a significant main effect for mortality salience [*F*(1, 235) = 5.72, *p* = .02]. Negative affect did not predict ad attitudes (*F* = 2.44, *p* = .12). Consistent with past research (Dahl et al. 2009; Griffitt and Kaiser 1978; LaTour and Henthorne 1993; Sengupta and Dahl 2008; Wyllie et al. 2014), male participants (*M* = 4.20, *SD* = 1.10) responded more favorably toward the ads than females (*M* = 4.01, *SD* = 0.86). Additionally, the non-sex appeal ad was evaluated more favorably overall (*M*_control_ = 4.26, *SD* = 0.85 vs. *M*_sex−appeal_ = 3.93, *SD* = 1.06). Further, participants in the mortality condition showed less favorable attitudes towards the ads than those under control condition (*M*_death_ = 3.96, SD = 0.99 vs. *M*_control_ = 4.25, *SD* = 0.94). The two-way interaction was significant between gender and sex appeal [*F*(1, 235) = 4.38, *p* = .04] but not between MS and sex appeal [*F*(1, 235) = 2.05, *p* = .15], or between MS and gender [*F*(1, 235) = 2.01, *p* = .16]. Most importantly, and consistent with our prediction, the three-way interaction was significant [*F*(1, 235) = 7.45, *p* = .01; see Fig. 2].

Post hoc testing supported our predictions such that males had more negative attitudes toward the sexualized advertisement when death was salient (*M* = 3.69, *SD* = 1.11) than in the control condition [*M* = 4.64, *SD* = 1.11; *F*(1, 235) = 16.47, *p* < .001]. However, females’ attitudes toward the sex appeal ad were not statistically different between mortality salience and control condition [*M*_death_ = 3.72, *SD* = 1.06 vs. *M*_control_ = 3.69, *SD* = 0.51; *F*(1, 235) = 0.03, *p* = .86]. For both men and women, views of the non-sexualized advertisement did not differ between mortality salience and the control condition (*p*s > 0.25).

#### Purchase intentions

We then conducted an an identical ANCOVA, except with intentions to purchase products as the dependent variable. There was a main effect of mortality salience [*F*(1, 235) = 10.14, *p* < .01] and a two-way interaction effect between gender and advertisement type [*F*(1, 235) = 3.89, *p* = .05]. Generally, participants indicated less likelihood to purchase products with death thoughts primed (*M*_death_ = 3.06, *SD* = 1.22 vs. *M*_control_ = 3.62, *SD* = 1.36). Males didn’t show any difference in the response to the ad type [*M*_sex−appeal_ = 3.48, *SD* = 1.49 vs. *M*_non sex−appeal_ = 3.40, *SD* = 1.27; *F*(1, 235) = 0.11, *p* = .74]. However, women disliked sexually appealing ads more than the non-sexualized ads [*M*_sex−appeal_ = 2.91, *SD* = 1.18 vs. *M*_non sex−appeal_ = 3.46, *SD* = 1.23; *F*(1, 235) = 5.93, *p* = .02]. More importantly, a significant three-way interaction effect emerged, *F*(1, 235) = 3.79, *p* = .05 (see Fig. 2).

Post hoc testing revealed that males’ purchase intentions when the product was presented in sexually appealing ads were significantly decreased when mortality was salient compared to the control condition [*M*_death/sex−appeal_ = 2.91, *SD* = 1.21 vs. *M*_control/sex−appeal_ = 4.11, *SD* = 1.54; *F*(1, 235) = 14.02, *p* < .001], while that of females was not [*M*_death/sex−appeal_ = 2.89, *SD* = 1.22 vs. *M*_control/sex−appeal_ = 2.95, *SD* = 1.17; *F*(1, 235) = 0.03, *p* = .87]. In contrast, for men and women views of the non-sexualized advertisement were not impacte by mortality salience (for men, *p* = .31, for women, *p* = .09).

#### Alternate analyses

All significant effects reported in Study 1 remained significant when overall negative affect was not entered as a covariate. All non-significant effects reported in this study remained non-significant as well without this covariate.

### Discussion

The results of Study 1 supported our hypothesis. Men became more negative towards sexualized advertisements featuring women when mortality was salient compared to the salience of a control topic. Further, this extended to the effectiveness of the ads, as men also reported reduced intentions to buy the products (vodka and perfume) if death was salient, when the advertisement featured sexualized women. In contrast, death salience had no impact on these responses for women. This research is consistent with past research indicating that men (but not women) view female sexuality more negatively when mortality is salient (e.g., Landau et al. 2006), including when these women are depicted in advertisements (Morris and Goldenberg 2015). It extends this research by demonstrating that mortality salience impacts views of advertisements associated with female sexualization, and the desire to purchase these products (and not just to views of the women themselves).

## Study 2

The first goal of Study 2 was to conceptually replicate Study 1. The second was to test a potential mediator—disgust—for the effect that mortality salience is having on increasing negative views of sexualized advertisements and reducing purchase intentions. We hypothesized that death-primed men would respond with greater disgust to ads featuring sexualized women, and that this would explain why they rate the advertisements and products more negatively. Thus, once death thoughts are activated, men will respond with disgust to sex appeal advertising, and their feeling of disgust would lead them to have negative attitudes toward the ads. This is consistent with recent research (Lambert et al. 2014) indicating that specific negative affective reactions (e.g., a single emotion) as opposed to broad indices of negative and positive affect (which were found not to mediate effects by a wide body of researchers; Burke et al. 2010) can mediate the effects of mortality salience (see also, Echebarria-Echabe and Perez 2015).

**Fig. 3:**
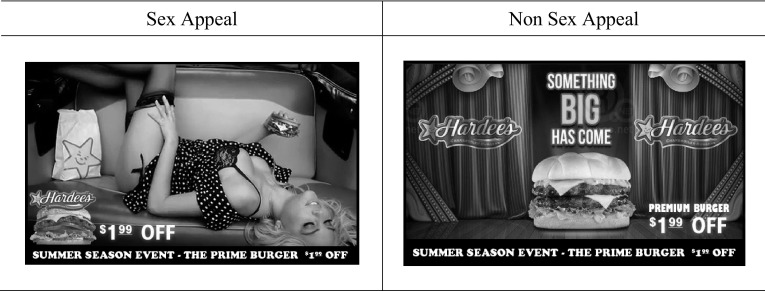
Study 2 advertising stimuli

In Study 2, we also wanted to test the effects using a product that is (probably) more typically purchased by Korean college students,^2^ and to use a product that is not as stereotypically linked to sexualization as vodka and perfume. Stereotyping and the desire for well-structured (e.g., linked) information increases when mortality is salient (Landau et al. 2004; Schimel et al. 1999) (note though that this link cannot account for the effects of Study 1 as the typicality between sexualization and the product/brand/advertisement did not differ between the mortality thought and the control condition, but results were still obtained). Thus, using hamburgers—a product not typically associated with sexualization- enables us to further test our hypotheses (see Fig. 3).

In addition, we also changed from a death-containing video clip to an online newspaper article containing the news of people committing suicide. This change could potentially enhance the external validity of our findings because many people are exposed to death-related news from online sources, and death related news occurs in many forms that are not directly related to war and conflict (as were the materials in Study 1). Indeed, only one study that we know of (Echebarria-Echabe and Perez 2015) has manipulated thoughts of death via information about suicides and it used pictures. Finally, Study 2 measured two dependent variables, advertising attitudes and purchase intention, which was assessed with a several item measure instead of the single item in Study 1.

### Method

#### Participants

Two hundred seventy-five Korean undergraduate students participated in the experiment in exchange for partial course credit (females = 143, males = 132). They were randomly assigned to a 2 (MS: Death vs. Control) x 2 (Gender: Male vs. Female) x 2 (Sex appeal: Yes vs. No) between-subject design, with all materials being presented in the Korean language.

#### Procedure and materials

The procedure and materials of Study 2 were identical to that of Study 1, with a few exceptions. To prime death-thoughts, this study used an online newspaper article about suicide. Participants in the control condition were asked to read an online newspaper article on the difficulty of getting a job after college graduation (consistent with dozens of past studies priming failure as a control condition to mortality salience; e.g., Landau et al. 2009). Participants were randomly assigned to read either the suicide-related article or the job-failure related article. Further, after the manipulation checks for death thought accessibility, participants were randomly exposed to either a sex appeal ad or non sex appeal ad for the hamburger brand of Hardee’s (see Fig. 3).

After the advertisement, participants were asked to indicate the extent of disgust, along with other feelings (liking, bad, disgust, fearful), that they felt while viewing the ad with options ranging from 1 (*not at all*) to 7 (*very much*; Shimp and Stuart 2004). Next, to measure ad attitudes and purchase intentions, Study 2 employed almost the same scales used in Study 1. However, one item (*bad—good*) of ad attitude measurement was added and reverse coded to ensure that respondents do not blindly mark all questions in the same direction. Ad attitudes were measured on three seven-point scales (1 = *poor*/*good*/*dislike*; 7 = *excellent*/*bad*/*like*; α = 0.67). Purchase intentions were also measured on three seven-point scales (1 = *no purchase intention*/*no recommendation*/*not purchase*; 7 = *high purchase intention*/*recommendation*/*definitely purchase*; α = 0.90). These measurements were averaged to make indexes for each variable.

### Results

#### Manipulation checks

We averaged the number of death-related words that each participant answered to create the death thought accessibility index. The index for participants under the MS condition were higher than that of those under the control condition, *F*(1, 272) = 63.67, *p* < .001; *M*_death_ = 2.57, *SD* = 1.92, vs. *M*_control_ = 1.01, *SD* = 1.25. Further, with the same measurement in Study 1, we compared the degree of sexiness of the advertising stimuli. The results were consistent with our manipulation, revealing a higher score for sex appeal (*M* = 7.93, *SD* = 2.13) than for control ad (*M* = 2.44, *SD* = 1.84), *F*(1, 273) = 524.42, *p* < .001.

#### Affect

Overall mood was not different in either mortality salience or control condition. The average score of positive mood when mortality was salient (*M* = 3.69, *SD* = 1.33) did not differ from that of control condition (*M* = 3.45, *SD* = 1.08), *F*(1, 273) = 2.54, *p* = .11. Unlike Study 1, the average score of negative mood was also not different between conditions (*M*_death_ = 3.18, *SD* = 1.18 vs. *M*_control_ = 3.07, *SD* = 1.08), *F*(1, 273) = 0.73, p = .39, so we did not choose to enter it as a covariate in subsequent analyses.

#### Ad attitudes

**Fig. 4:**
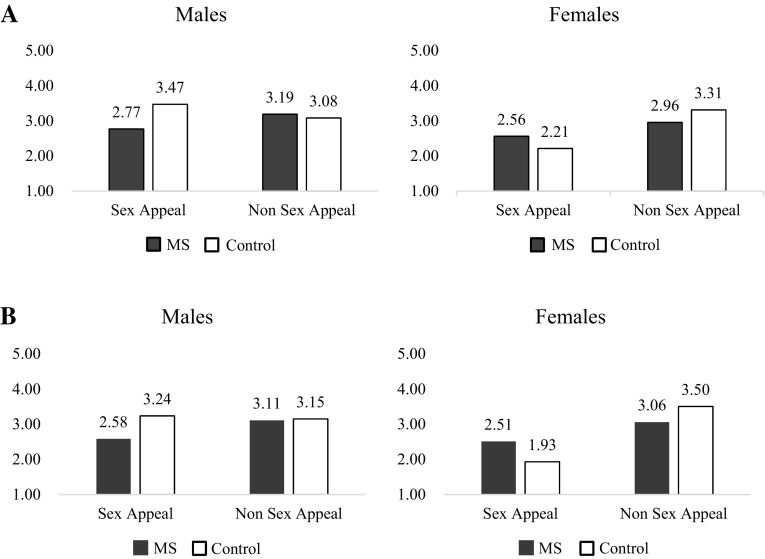
Sex appeal ad attitudes (**a**) and purchase intention (**b**) for study

A three-way ANOVA revealed two significant main effects, one for gender [*M*_male_ = 3.14 vs. *M*_female_ = 2.76; *F*(1, 267) = 5.01, *p* = .03] and one for sex appeal [*M*_sex−appeal_ = 2.74 vs. *M*_non sex−appeal_ = 3.14; *F*(1, 267) = 5.42, *p* = .02]. The two-way interaction between gender and sex appeal, *F*(1, 267) = 4.93, *p* = .03, was also significant. The two-way interaction effect indicated that male participants reported no differences in attitudes between the sexually appealing (*M* = 3.14, *SD* = 1.64) and control ads (*M* = 3.14, *SD* = 1.37), *F* < 1, *p* = .98, whereas female participants showed a significantly less favorable attitude towards sexually appealing ads (*M* = 2.38, *SD* = 1.28) compared to control ads (*M* = 3.14, *SD* = 1.20), *F*(1, 267) = 11.02, *p* = .001. More importantly to our hypotheses, the three way interaction was significant, *F*(1, 267) = 5.31, *p* = .02 (see Fig. 4).

Post hoc testing indicated that males’ attitudes toward the sex appeal ad were significantly decreased under mortality salience (*M* = 2.77, *SD* = 1.55) compared to the control condition (*M* = 3.47, *SD* = 1.67), *F*(1, 267) = 4.23, *p* = .04. Meanwhile, females’ attitudes toward the sex appeal ad were not significantly different between the two conditions (*M*_death_ = 2.56, *SD* = 1.38, *M*_control_ = 2.21, *SD* = 1.17), *F*(1, 267) = 1.16, *p* = .28. Additionally, men’s and women’s views of the non-sexualized advertisement did not differ between the mortality condition and the control conditions (*p*s > 0.25).

#### Purchase intention

A three-way ANOVA on purchase intention found three effects reaching significance: a main effect of sex appeal [*M*_sex−appeal_ = 2.55 vs. *M*_non sex−appeal_ = 3.21, *F*(1, 267) = 13.68, *p* < .001], a two-way interaction between gender and sex appeal [*F*(1, 267) = 5.87, *p* = .02], and the predicted three-way interaction among mortality salience, gender, and sex appeal [*F*(1, 267) = 5.64, *p* = .02; see Fig. 4]. A significant two-way interaction indicated that males’ purchase intentions for a product were not significantly different between the sexualized and non-sexualized advertisements (*M*_sex−appeal_ = 2.93, *SD* = 1.64 vs. *M*_non sex−appeal_ = 3.13, *SD* = 1.55), *F*(1, 267) = 0.64, *p* = .42, whereas females reported a significantly less intention to purchase the product presented in sexually appealing ads (*M* = 2.22, *SD* = 1.32) than the neutral ads (*M* = 3.29, *SD* = 1.28), *F*(1, 267) = 19.92, *p* < .001.

Post hoc testing relating to the three way interaction revealed that males’ intention to purchase the target product when the advertisement featured female sexualization marginally decreased in the death thought condition (*M* = 2.58, *SD* = 1.42) compared to the control condition (*M* = 3.24, *SD* = 1.77), *F*(1, 267) = 3.33, *p* = .07. On the other hand, females’ intention to purchase the target product featured in sex appeal ad were directionally increased under mortality salience condition (*M* = 2.51, *SD* = 1.57) than control condition (*M* = 1.93, *SD* = 0.94), *F*(1, 267) = 2.97, *p* = .09. Further, attitudes toward the non-sexualized essay did not differ for men and women between mortality salience and the control condition (*p*s > 0.18).

#### Mediation analyses

We next wanted to test if disgust mediated the effects of mortality salience on men’s negative reactions to the advertisements (Hayes 2013).

Across all participants in all conditions, pearson correlations indicated that disgust was negatively associated with both attitudes toward the advertisement (*r* = −.263, *p* < .001) and purchase intentions (*r* = −.361, *p* < .001). PROCESS (Model 3) was then used to test the impact of mortality salience (1 = Mortality Thoughts, 2 = Control Thoughts), Gender (1 = Men, 2 = Women) and Advertisement Type (1 = Sexual, 2 = Non-Sexual) on disgust. The three way interaction was significant, *b* = −1.59, *SE* = 0.69, *t* = −2.31, *p* = .02. Within this, the Advertisement Type X Mortality Salience interaction was significant for men, *b* = 1.02, *SE* = 0.49, *t* = 2.05, *p* = .04, but not for women, *b* = −0.57, *SE* = 0.48, *t* = −1.18, *p* = .22. Further analyses indicated that for men in the sexualized condition, mortality salience was associated with more disgust, *b* = −1.28, *SE* = 0.357, *t* = −3.58, *p* < .001, but that mortality salience was unrelated for men in the non-sexualized condition (*p* = .46), and for women in either the sexualized or the non-sexualized conditions (*p*s > 0.17).

As disgust negatively predicted advertisement attitudes and purchase intentions, and was heightened by mortality salience only for men within the sexualized advertisement condition, we next conducted moderated-mediation analyses (Model 8) using only male participants. Conditional analyses indicated that disgust was a significant mediator in the sex condition for advertisement attitudes (indirect effect = 0.29; LLCI = 0.0563, ULCI = 0.7451) and for purchase intentions (indirect effect = 0.32; LLCI = 0.0826, ULCI = 0.7671). As such, heightened disgust mediated the effect of mortality salience on both advertisement attitudes and purchases intentions within the sexualized advertisement.

#### Additional analyses

The three way interaction for purchase intentions and for ad attitudes remained significant with negative affect (assessed using the PANAS, which does not include disgust) also included as a covariate. Additionally, the mediation analysis revealed that the specific emotion of disgust mediated the effects even with overall negative affect included. Thus, disgust, and not an overall index of negative affect, mediated the effect of mortality salience on attitudes towards sex-appeal advertisements and purchase intentions for men.

### Discussion

Consistent with Study 1 and our hypotheses, Study 2 revealed that death reminders in media contexts increase males’ negative evaluation of sex appeal ads, and reduces their intentions to buy products associated with those advertisements compared to a control topic. Women, however, showed no similar effect or, in the case of purchase intentions, showed an opposite (marginal) effect when death was salient for the sexualized ads. Further, the results showed that disgust while viewing the ad mediates the effect of death thoughts on men’s evaluations on sex appeal ads and their purchase intentions.

## General discussion

Two experimental studies tested, for the first time, the impact of mortality salience on how people evaluate products and advertisements that feature female sexualization. Consistent with our hypotheses derived from TMT (Greenberg et al. 1990), men, but not women, were more negative towards advertisements featuring female sexuality, and were less likely to purchase the advertised products, when mortality was salient. This occurred across three products using two different manipulations of mortality salience. Further, in Study 2, we found evidence that disgust mediated men’s responses to advertisements featuring female sexuality; mortality salience increased their disgust, which in turn was associated with more negative reactions.

### Terror management theory

These studies have important implications for understanding terror management processes. First, they extend past research which focused specifically on behaviors and attitudes toward sexualized women as a function of mortality salience, to views of products and brands that utilize female sex-appeal and purchase intentions. Second, these studies build on a growing body of evidence (Echabarra-Echabe and Perez 2015; Lambert et al. 2014) that specific emotions mediate terror management effects (as opposed to broad indices of positive and negative affect, which were found over hundreds of published studies—and the current studies- to not mediate effects; Lambert et al. 2014). However, the specific emotion seems to differ based on the response to mortality thoughts (e.g., fear for self-esteem, but not for worldview defense, Lambert et al. 2014; disgust for reactions to sexualized women in ads, in Study 2). This, in conjunction with a wide range of research demonstrating that mortality salience primes can impact specific emotions proximally (Lambert et al. 2014) and distally (Routledge et al. 2010; Wisman and Heflick 2016), that terminal diagnoses cause intense, complex, emotional reactions (Gum and Snyder 2002; Yun et al. 2010), and that emotional arousal is necessary for mortality salience to impact worldview defense (Webber et al. 2015), suggests that past experimental research, and the conclusions derived from them arguing that mortality has effects due to purely cognitive reasons (Hayes et al. 2010), may have underestimated the importance of emotion in terror management processes.

### Marketing implications

This research builds on past research in marketing and consumer behavior in several ways. Consistent with past research, men tended to prefer sexualized advertisements (Dahl et al. 2009; LaTour 1990; LaTour and Henthorne 1993; Sengupta and Dahl 2008; Wyllie et al. 2014), whereas women did not. This extended to the desire to purchase the products associated with those advertisements. Importantly, these studies identify one key factor—thoughts of death—that caused men to have more negative attitudes toward sexualized ads, and reduced intentions to buy the associated products (compared to when death was not primed). Additionally, this research builds on past work exploring the impact of mortality salience on consumer preferences by examining views of products and advertisements, as opposed to solely testing consumer behavior (Ferraro et al. 2005; Huang and Wyer 2015). Studies 1 and 2 also demonstrate that both print media and video media can prime mortality, and in doing so, impact views of advertisements.

This work also builds on research on sexualization, dehumanization and consumer behavior. Bongiorno and colleagues (e.g., Bongiorno et al. 2013) found that for animal charities, advertisements featuring sexualized women have negative effects. This was mediated by the belief that the women in the advertisements were more animalistic (i.e., less human) when sexualized. As disgust has been posited to function to delineate humanness from animality (Rozin and Fallon 1987), this suggests—like the current research (in which disgust was heightened towards sexualized ad when death was primed)—that the perceived humanness of people in advertisements plays an important role in how people evaluate advertisements as a whole, and when death is salient.

### Limitations and alternative explanations

We made a gender-specific hypothesis suggesting that mortality salience would only impact views of sexualized advertisements featuring women for men, but we did not test views of adverts featuring male sexuality. This was due partially to the already complex effects (three way interactions) we predicted and measured, and due to past research indicating that views of men when sexualized are not altered when mortality is salient (Landau et al. 2006).

Yet another limitation of the current research is that we did not measure how long these effects last. Though most TMT research has found that longer delays (in the context of a lab study session) increase the effect size of mortality salience effects (Martens et al. 2011), it remains very much unknown how the impact of mortality salience plays out over long periods of time (e.g., weeks), both within and outside of the laboratory.

In both studies, the sexualized women advertisements featured a person whereas the control advertisements did not. The non-sexualized advertisements were chosen in part because they are real advertisements that have been used. While this lack of a person in these ads is a limitation, it does not seem likely that using control advertisements with non-sexualized women would have impacted our pattern of results. Indeed, Landau et al. (2006) found that “wholesome” women are not denigrated more when mortality is salient (relative to a control condition) by men, while finding that sexualized women are.

Another possible consideration is that the advertisements were for foreign (non-Korean) companies. It is true that mortality salience motivates in-group favoritism and in-group humanization when mortality is salient in Western Cultures (e.g., Vaes et al. 2010). But, in each study, the advertisements were foreign in both the mortality salience and control conditions, and across the sexualized and non-sexualized conditions. As such, if thoughts of death were simply causing out-group derogation, we would expect a main effect of mortality salience in reducing liking for both the sexualized advertisement and the non-sexualized advertisement across genders (compared to the control condition). Further, in Eastern cultures we might even expect mortality salience to promote less negativity towards out-groups (Feng et al. 2016). We found, however, that mortality salience reduced positive evaluations only for the sexualized advertisement, and only for men. This pattern of results fits neither of these possibilities.

Another possible alternative explanation for these results is that the advertisements were perceived as immoral, and/or disgusting, and that mortality salience simply was inducing worldview defense as it has done in dozens of studies (Burke et al. 2010). Women, however, tend to hold more negative views of sexualize advertisements in general (which we also found in relation to the non-sexualized advertisements). In turn, if this were true, we would expect women, but not men, to become more negative towards sexually alluring advertisements when mortality is salient. This is the opposite of what was found.

## Conclusion

There is a high prevalence of using female sexuality with the intention to sell across a wide range of products, entertainment types (e.g., sports) and media. While men typically enjoy advertisements that utilize sex appeal, and female sexuality, the current research suggests that, in conjunction with thoughts of death (which are primed in many media contexts, such as television programs, movies, internet articles, and also in the physical environment, such as cemeteries, hospitals, war memorials, funeral homes), these advertisements would not be effective as a means to sell products. On a broader level, they suggest that, in the context of existential concerns, sexuality, and in particular female sexuality, continues to be paradoxical, serving as a key component to the origin of human life and as a source of immense pleasure, but remaining a lens through which we are disgusted with our own animal nature.

## Appendix

### Items used for mortality manipulation check

Instruction: Please complete the words. Each is either 2- or 3-letter words if not specified otherwise.

1. ______음: supposed to be죽음(death) vs. 화음(harmony).
2. ______체: supposed to be 시체 or 사체 (dead body) vs. 신체(body).
3. 장______ (two-letter): supposed to be 장례 vs. 장래(future).
4. ______망: supposed to be 사망(death) vs. 희망(hope).
5. ______해: supposed to be 상해(injury) or 재해(disaster) vs. 새해(new year).
6. 무______: supposed to be 무덤(grave) vs. 무료(free).
7. 장______(three-letter): supposed to be 장례식(funeral) vs. 장난감(Toy).
8. ______례: supposed to be 장례(funeral) vs. 무례(rudeness).
9. 천______: supposed to be 천국(heaven) vs. 천막(tent).
10. 화______: supposed to be 화장(cremation) vs. 화분(flower pot) or 화요일(Tuesday).

## Footnotes

1 Morris and Goldenberg (2015) found that mortality salience caused men to distance from a sexualized woman, but not if she was“objectified.” In this research, objectified meant being merged in an image with an actual physical object (it did not mean being sexualized). As such, our manipulations of sexulization in Studies 1 and 2 were akin to their control condition in which women were not objectified; we did not have a condition that mirrored their objectified condition.

2 Controlling for familiarity with the advertisements did not impact the significant pattern of results across all analyses.
